# Children’s Health in London and Luton (CHILL) cohort: a 12-month natural experimental study of the effects of the Ultra Low Emission Zone on children’s travel to school

**DOI:** 10.1186/s12966-024-01621-7

**Published:** 2024-09-05

**Authors:** Christina Xiao, James Scales, Jasmine Chavda, Rosamund E. Dove, Ivelina Tsocheva, Helen E. Wood, Harpal Kalsi, Luke Sartori, Grainne Colligan, Jessica Moon, Esther Lie, Kristian Petrovic, Bill Day, Cheryll Howett, Amanda Keighley, Borislava Mihaylova, Veronica Toffolutti, Jonathan Grigg, Gurch Randhawa, Aziz Sheikh, Monica Fletcher, Ian Mudway, Sean Beevers, W. James Gauderman, Christopher J. Griffiths, Esther van Sluijs, Jenna Panter

**Affiliations:** 1 MRC Epidemiology Unit, School of Clinical Medicine, University of Cambridge, Box 285, Cambridge, UK; 2 Centre for Primary Care, Wolfson Institute of Population Health, Barts and the London School of Medicine and Dentistry, Queen Mary University of London, London, UK; 3 Asthma UK Centre for Applied Research, Edinburgh, UK; 4 Institute for Health Research, University of Bedfordshire, Luton, UK; 5 Social Action for Health, London, UK; 6 Centre of the Cell, Queen Mary University of London, London, UK; 7 Health Economics and Policy Research Unit, Wolfson Institute of Population Health, Queen Mary University of London, London, UK; 8 Nuffield Department of Population Health, University of Oxford, Oxford, UK; 9 Blizard Institute, Faculty of Medicine and Dentistry, Queen Mary University of London, London, UK; 10 Usher Institute, University of Edinburgh, Edinburgh, UK; 11 MRC Asthma UK Centre in Allergic Mechanisms of Asthma, London, UK; 12 MRC Centre for Environment and Health, Imperial College London, London, UK; 13 NIHR Health Protection Research Units in Environmental Exposures and Health, and Chemical and Radiation Threats and Hazards, Imperial College London, London, UK; 14 Keck School of Medicine, University of Southern California, Los Angeles, USA

**Keywords:** Active travel, Children’s health, Health policy, Clean air zones, Natural experiment

## Abstract

**Background:**

The Ultra-Low Emission Zone (ULEZ), introduced in Central London in April 2019, aims to enhance air quality and improve public health. The Children's Health in London and Luton (CHILL) study evaluates the impact of the ULEZ on children's health. This analysis focuses on the one-year impacts on the shift towards active travel to school.

**Methods:**

CHILL is a prospective parallel cohort study of ethnically diverse children, aged 6–9 years attending 84 primary schools within or with catchment areas encompassing London’s ULEZ (intervention) and Luton (non-intervention area). Baseline (2018/19) and one-year follow-up (2019/20) data were collected at school visits from 1992 (58%) children who reported their mode of travel to school ‘today’ (day of assessment). Multilevel logistic regressions were performed to analyse associations between the introduction of the ULEZ and the likelihood of switching from inactive to active travel modes, and vice-versa. Interactions between intervention group status and pre-specified effect modifiers were also explored.

**Results:**

Among children who took inactive modes at baseline, 42% of children in London and 20% of children in Luton switched to active modes. For children taking active modes at baseline, 5% of children in London and 21% of children in Luton switched to inactive modes. Relative to the children in Luton, children in London were more likely to have switched from inactive to active modes (OR 3.51, 95% CI 1.68–7.31). Children in the intervention group were also less likely to switch from active to inactive modes (OR 0.22, 0.12–0.41). Moderator analyses showed that children living further from school were more likely to switch from inactive to active modes (OR 5.20; 1.67–15.21) compared to those living closer (OR 1.54, 0.33-7.21).

**Conclusions:**

Implementation of clean air zones can increase uptake of active travel to school and was particularly associated with more sustainable and active travel in children living further from school.

**Supplementary Information:**

The online version contains supplementary material available at 10.1186/s12966-024-01621-7.

## Background

Motorised vehicle use negatively impacts health throughout life, influencing children's physical activity, sedentary behaviour, and contributing to diseases like childhood asthma linked to air pollution. Regular physical activity, crucial for children's healthy growth and mental well-being [1, 2], also plays a vital role in preventing the development of obesity [3], prevalent in 23% of children aged 10–11 in 2022 [4]. Despite UK guidelines recommending 60 min of average daily moderate-to-vigorous physical activity for children aged 5–18 [5], only 45% of children aged 5 to 16 met these levels in 2021 [6].

Active travel to school, such as walking, cycling, or scootering, can provide routine physical activity, helping achieve recommended levels [7]. However, from 2005 to 2021, the proportion of trips taken by private vehicles to school increased from 31 to 37% among urban primary school children in England, while walking trips decreased from 48 to 45% [8]. A similar decline in active travel to school has been observed in other countries [9].

Replacing motorised trips to school with active travel may also reduce air pollution, which is a major risk factor for non-communicable diseases and one of the leading causes of mortality globally [10]. Children, due to their ongoing organ development, time spent outdoors, and higher breathing rates relative to body mass, are more vulnerable to the impacts of traffic-related air pollution than adults [11]. Increased childhood exposure to traffic-related air pollution has been found to affect neurodevelopment, cognitive ability, and lung function [12]. In addition, exposure at a young age can predispose children to lung impairment later in life [13].

Policies that aim to reduce motorised vehicle traffic can be part of an overall strategy to decrease exposure to air pollution for children, promote active travel, and reduce barriers to physical activity. Clean air zones (CAZ) are an example of a policy aiming to reduce motorised vehicle use, different types of air pollutants (e.g., NO_2_ and the traffic derived component of PM_2.5_), and greenhouse gases (e.g., CO_2_). Implemented in over 300 European cities as a major component of traffic emission reduction strategies [14], these interventions aim to alter transportation behaviour by limiting access to the most polluting motorised vehicles or imposing financial disincentives in defined geographical areas [15, 16].

Studies on CAZs have examined their impact on air pollution levels and related health outcomes, finding consistent evidence of reductions in cardiovascular disease following the introduction of these schemes [17]. However, studies on CAZs’ effects on children’s health are limited, with one finding no decrease in the proportion of children with smaller lungs despite reduced NO_2_levels [18]. Hypothesised pathways through which CAZs may affect children’s health include encouraging a shift to more active travel modes, reducing private vehicle use, or increasing the number of vehicles which meet the new emission standards (see Appendix Fig. 1 for an overview). However, the most health-promoting behaviour of interest may be the shift to active travel modes, which can both reduce air pollution emissions and replace sedentary behaviour with physical activity. Thus far, only one study has examined the effects of a CAZ on shifts in mode of transport, finding that 60% of former private vehicle users shifted to more active modes [16]. However, no studies have evaluated the effects of CAZs on children’s active travel.

The London Ultra Low Emission Zone (ULEZ) introduced into Central London in April 2019 provided the opportunity to perform a natural experimental evaluation to assess the effect of this policy on children’s mode of travel to school. We hypothesised that implementing the ULEZ in London would encourage children and their parents or carers to switch from inactive to active travel, while preventing a shift from active to inactive travel to school.

## Methods

### Study design and data

The Children’s Health in London and Luton (CHILL) study is a prospectively designed longitudinal study involving four years of data collection, with baseline data collection undertaken from June 2018 to April 2019, prior to ULEZ implementation in April 2019. This analysis reports on data from the baseline (June 2018-April 2019) and one-year follow-up prior to Covid-related school closures (June 2019-March 2020). Data were collected on a rolling basis, with a mean ± standard deviation [SD]: 12 ± 1 month interval between baseline and follow-up. At follow-up, mean ± SD exposure to the ULEZ was 7 ± 2 months.

**Fig. 1:**
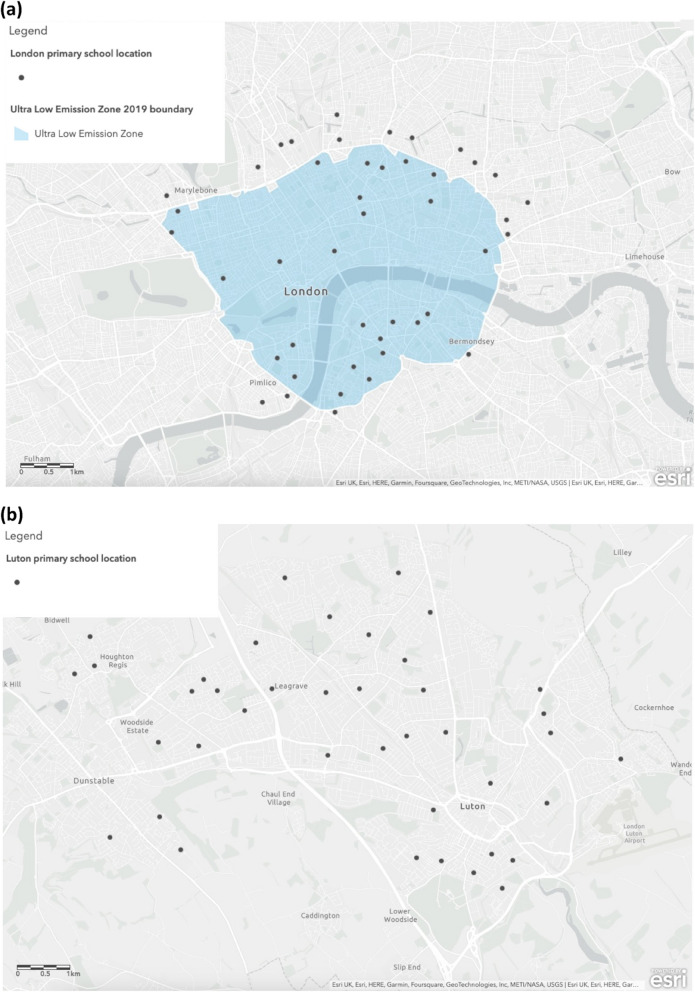
Location of primary schools in (**a**) London, the intervention site, and (**b**) Luton, the comparison site

The intervention group included schools with catchment areas within or bordering the Central London ULEZ (44 schools, representing 67% of those invited) (Fig. 1). Schools in the Borough of Luton were selected for the control group, with additional schools from the contiguous neighbouring town of Dunstable being recruited to reach a sufficient number of participants (32 in Luton and 8 in Dunstable, representing 71% of those invited; the power calculations used to determine the study sample size can be found in a previous study) [19]. The control group will be referred to as Luton for simplicity. Schools meeting the study criteria were directly approached, initially by the Chief Investigator or Site Lead, followed up by a member of the local research team (i.e. London or Luton), and invited to participate. Following agreement, meetings were held with head teachers or delegates to discuss details and address concerns. Recruiting students was the second stage of the recruitment process, with whole year groups being approached and children recruited from these schools (*n* = 84) if they were in year groups two, three, or four (aged six-nine years). Parental consent was mandatory for all child participants, and it was found that school assemblies, playground visits, classroom talks, and school communication channels were the most effective recruitment methods.

Study recruitment information and parental consent forms were sent home in school bags to be completed and returned to school prior to baseline. A trained field team visited each school for yearly health assessments to collect data on children’s lung function and questionnaire-based data. Parental questionnaires were sent home in school bags for parents to complete prior to the visit. Ethical approval was granted by the Queen Mary University of London Ethics Committee (reference: QMERC 2018/08). All participants’ parents or carers gave written consent; all children provided verbal assent at assessment. The protocol and further details describing this study’s methods and data collection have been described elsewhere [20].

### Intervention

The ULEZ was introduced in Central London on April 8, 2019, as an environmental and public health policy intervention to reduce traffic-related pollution and improve public health. All vehicles that did not meet specified exhaust emission standards (Euro Six for NO_x_ and PM_2.5_ from diesel vehicles, Euro Four for NO_x_from petrol vehicles and Euro Three for motorcycles/mopeds etc.) were subject to a daily charge to travel within the zone, with the scheme operating 24 h a day and 365 days per year [21]. The original ULEZ area was bounded by the Inner Ring Road and included the City of London and eight adjacent boroughs. It was extended in October 2021 to the north and south circular roads. Luton was chosen as the comparison area as Luton had a similar baseline air quality, demographics, and levels of socio-economic deprivation as the London ULEZ area. Moreover, Luton was chosen as there were no plans to introduce a charging scheme based on vehicle emission class during the study period and it was sufficiently distant to avoid the risk of contamination by the effects of the London ULEZ.

### Outcome

During annual health assessments at baseline and follow-up, children were asked ‘How did you travel to school today?’ (representing the day of the annual health assessment) and ‘How do you usually travel to school?’ (Appendix Table 1). Participants could choose one or more of the following transport mode categories: walking, cycling, scootering, bus, train/tube, private vehicles, taxi, or other. The validity and reliability of self-reported transport to school today by children aged 8–11 years have been reported to be high and has shown substantial agreement with parental reports on how the child travelled to school that day [22]. The mode of travel ‘today’ or ‘usually’ was then converted into two separate binary variables representing either active or inactive modes of transport. Active modes were classified as those that involved walking, cycling, or scootering during any part of the route or modes which included public transport (i.e., bus or train/tube), regardless if they also reported taking a private vehicle or taxi. Public transport was included as walking or cycling may be used to access it, even if no walking and cycling was reported [23]. Inactive modes were exclusively taking a private vehicle or taxi to school.

### Covariates

Parents reported on their employment and occupation status, household vehicle ownership at follow-up, residential address, and child demographics (age, sex, ethnicity) (Appendix Table 2). Using the 2019 English indices of deprivation tool [24], household deprivation and neighbourhood crime levels were determined based on residential postcodes. The measure used, Income Deprivation Affecting Children Index (IDACI), represents the proportion of children in income-deprived families. Both IDACI and crime measures were segmented into quintiles, with higher values indicating lower deprivation and crime levels. Using the 'gmapsdistance' package in R [25], the walking distance to school was calculated based on the child's residential and school addresses. This value was transformed into a binary variable using a 0.86-km cut-off, representing the median home-to-school distance within the sample. This value aligns with distances associated with increased likelihood of active school travel for children of a similar age in urban areas [26, 27].

### Data analysis

Descriptive data analysis was performed on children residing in London or Luton, with differences tested using independent samples t-tests for continuous variables or Pearson's χ2 tests for categorical variables. Crude, adjusted, and multilevel binomial logistic regressions, accounting for clustering of children within schools, were conducted to estimate associations between intervention group status and changes in school travel mode (switching from inactive travel at baseline to active travel at follow-up, or vice-versa). Models were adjusted for characteristics that were selected a-priori and included age, ethnicity (White or Black, Asian, and Minority Ethnic (BAME)), sex (male or female), parental employment status (full time, part time, unemployed, other), parental occupation (professional/managerial, skilled, unskilled, other), distance to school (0.86 km or > 0.86 km), vehicle ownership (yes or no), and neighbourhood-level deprivation and crime quintiles. In addition, we examined possible interaction effects by age, sex, ethnicity, distance to school, and vehicle ownership, as these variables were hypothesised to moderate the relationship between living and attending schools within the ULEZ and change in mode of travel to school [28].

We encountered convergence issues in the fully adjusted multilevel model for switching from active to inactive modes, likely due to overparameterization. To resolve this, we removed the 'parental occupation' variable, as socioeconomic factors were already captured by other covariates (parental employment, crime, IDACI). Sensitivity analyses showed that this exclusion did not significantly affect model fit (likelihood ratio test p = 0.964) and improved AIC and BIC values (Appendix Table 3), indicating a better-fitting, more parsimonious model.

We conducted two additional sensitivity analyses. Firstly, travel to school ‘today’ was the primary outcome to reduce recall bias but this may not accurately reflect habitual transport behaviour, so we also explored the results using usual travel to school as an outcome. Secondly, our primary analysis assumed that any trip involving active travel or public transport was an active trip, regardless of whether the child also reported travelling by private vehicle or taxi. As it was unknown whether the active or inactive mode comprised the majority of the trip, we also conducted a sensitivity analysis for a new modal shift variable considering any private vehicle or taxi usage as an inactive trip, even if children reported using other active modes.

Statistical significance was assumed at the five-percentage level. All statistical analyses were performed using R version 4.0.4 [29].

## Results

### Participants

**Fig. 2:**
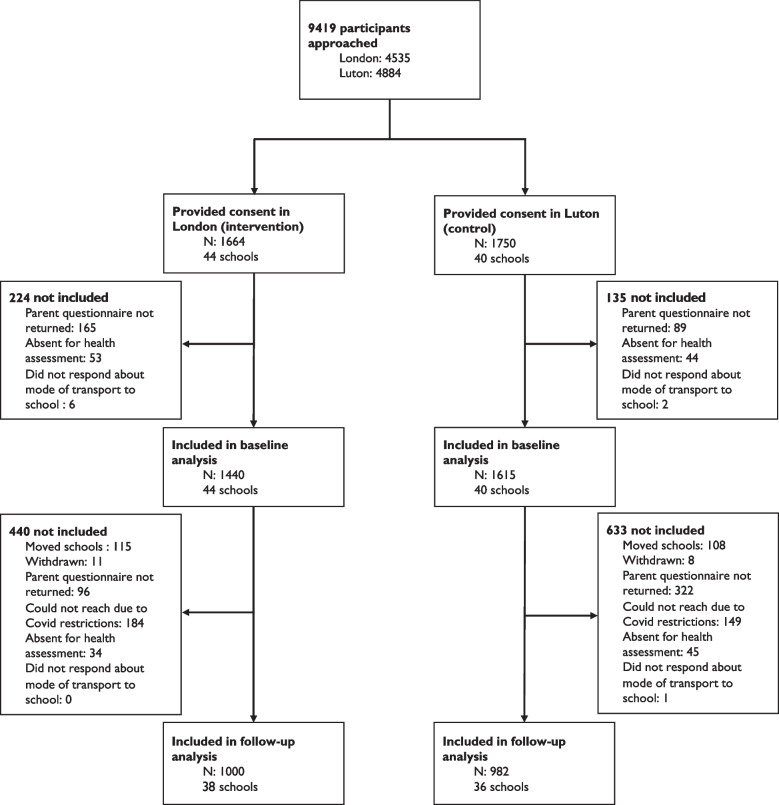
Study flow chart of participants included in the study

All children attending the recruited schools (*n* = 84) in year groups two, three, and four were eligible to participate (*n* = 9419). Written parental consent was obtained for 3414 (36%) children (Fig. 2). Of these, 1440 (87%) and 1615 (89%) children from London and Luton, respectively, returned the parental questionnaire, participated in the annual health assessment, and provided data on travel mode. At follow-up, 1000 (69%) children from London and 982 (61%) from Luton were retained. Student-level reasons for loss to follow-up included the child being absent during school visits (*n* = 79), the child moving schools (*n* = 223) and their parents withdrawing them from the study (*n* = 19). In addition, parental surveys (*n* = 418) were not returned at follow-up. School closures due to Covid-19 restrictions prevented follow-up data collection in ten schools (*n* = 323).

Children who were not included in the analysis at any time point (reasons for exclusion can be found in Fig. 2) from the London cohort (n = 664) were more likely to be male (48.5% vs. 42.4%), from a minority ethnic background (70.1% vs. 66.3%), more likely to have an ‘Other’ occupation (26.8% vs. 18.7%), and lived in areas with higher crime and neighbourhood deprivation compared to children living in London who were included in the analyses (Appendix Table 4). Luton children not included in the analyses (n = 768) were more likely to be older (7.9 vs. 7.7 years) at baseline, less likely to have parents in full-time employment (28.9% vs. 34.9%), more likely to live further from (58.9% vs. 51.7%) and lived in areas with higher levels of crime and neighbourhood deprivation than children living in Luton who were included in the analyses.

### Descriptive statistics

Table 1 presents the study participants’ demographic characteristics. Children who lived in London were slightly older with a mean age of 7.9 (SD 0.9) compared to Luton, which had a mean age of 7.7 (SD 0.9). In London, participants were more likely to be female (*p* = 0.002), of a minority ethnic background (*p* < 0.001), have an unemployed or parent with other employment status (*p* = 0.038), have a parent with professional or managerial occupations (*p* = 0.007), live closer to school (*p* = 0.008), live in a household without a private vehicle (*p* < 0.001), and reside in areas with lower levels of crime (*p* < 0.001) and higher levels of deprivation (*p* < 0.001) compared to children living in Luton. These statistically significant differences were accounted for in analyses.

**Table 1:** Descriptive baseline characteristics of the study population^*^

Covariate	London (n = 1000)		Luton (n = 982)		*p*-value*
Age (mean, SD)					
Baseline	7.9	(0.9)	7.7	(0.9)	<0.001
Follow-up	8.9	(0.9)	8.7	(0.7)	<0.001
Sex (n, %)					
Male	424	(42.4)	490	(49.9)	0.002
Female	576	(57.6)	492	(50.1)	
Ethnicity (n, %)					
BAME	629	(66.3)	572	(59.8)	<0.001
White	320	(33.7)	384	(40.2)	
Employment status (n, %)					
Full-time	279	(32.2)	317	(34.9)	0.038
Part-time	224	(25.9)	231	(25.4)	
Unemployed	126	(14.5)	119	(13.1)	
Other	237	(27.4)	241	(26.5)	
Occupational category (n, %)					
Professional/Managerial	368	(56.0)	310	(45.7)	0.007
Skilled	96	(14.6)	112	(16.5)	
Unskilled	70	(10.7)	79	(11.7)	
Other	123	(18.7)	177	(26.1)	
Distance to school (n, %)					
Near (≤0.86 km)	**475**	**(51.3)**	**351**	**(48.3)**	**0.254**
Far (>0.86 km)	**451**	**(48.7)**	**375**	**(51.7)**	
Vehicle ownership** (n, %)					
Yes	461	(54.1)	798	(89.7)	<0.001
No	391	(45.9)	92	(10.3)	
Crime Quintile (n, %)					
1 (highest crime)	**309**	**(31.0)**	**302**	**(30.9)**	<0.001
2	**301**	**(30.2)**	**328**	**(33.6)**	
3	**172**	**(17.2)**	**237**	**(24.3)**	
4	**115**	**(11.5)**	**89**	**(9.1)**	
5 (lowest crime)	**101**	**(10.1)**	**21**	**(2.1)**	
IDACI Quintile (n, %)					
1 (highest level of deprivation)	**578**	**(57.9)**	**190**	**(19.4)**	<0.001
2	**269**	**(27.0)**	**368**	**(37.7)**	
3	**75**	**(7.5)**	**282**	**(28.9)**	
4	**34**	**(3.4)**	**114**	**(11.7)**	
5 (lowest level of deprivation)	**42**	**(4.2)**	**23**	**(2.4)**	

Sums of the number of participants with each characteristic may equal the total number of participants if data is missing

*n* Number, *BAME* Black, Asian, and Minority Ethnic, *SD* Standard deviation, *km* Kilometre, *IDACI* Index Deprivation Affecting Children Index

^*^p value refers to independent samples t-tests for continuous variables or Pearson's χ2 tests for categorical variables

^**^Vehicle ownership data was only collected at follow-up

Table 2 presents the number of children taking active or inactive modes of transport in London and Luton at baseline and follow-up. Among children who were active at baseline, a greater proportion of children remained active in London (95%) compared to in Luton (79%), and fewer switched to inactive modes (5%) compared to in Luton (21%). Among children who were inactive at baseline, a greater proportion of children switched to active modes in London (42%) compared to Luton (20%). Most children (80%) maintained their use of inactive modes in Luton, compared to 58% in London.

**Table 2:** Proportion of children maintaining or switching modes in London and Luton

Baseline	Follow-up	Group	London	Luton
			n (%)	n (%)
Active	Active	Maintained active modes	812 (94%)	475 (79%)
	Inactive	Switched to inactive modes	48 (6%)	124 (21%)
Inactive	Active	Switched to active modes	44 (42%)	74 (20%)
	Inactive	Maintained inactive modes	61 (58%)	290 (80%)

### Switching travel modes

**Fig. 3:**
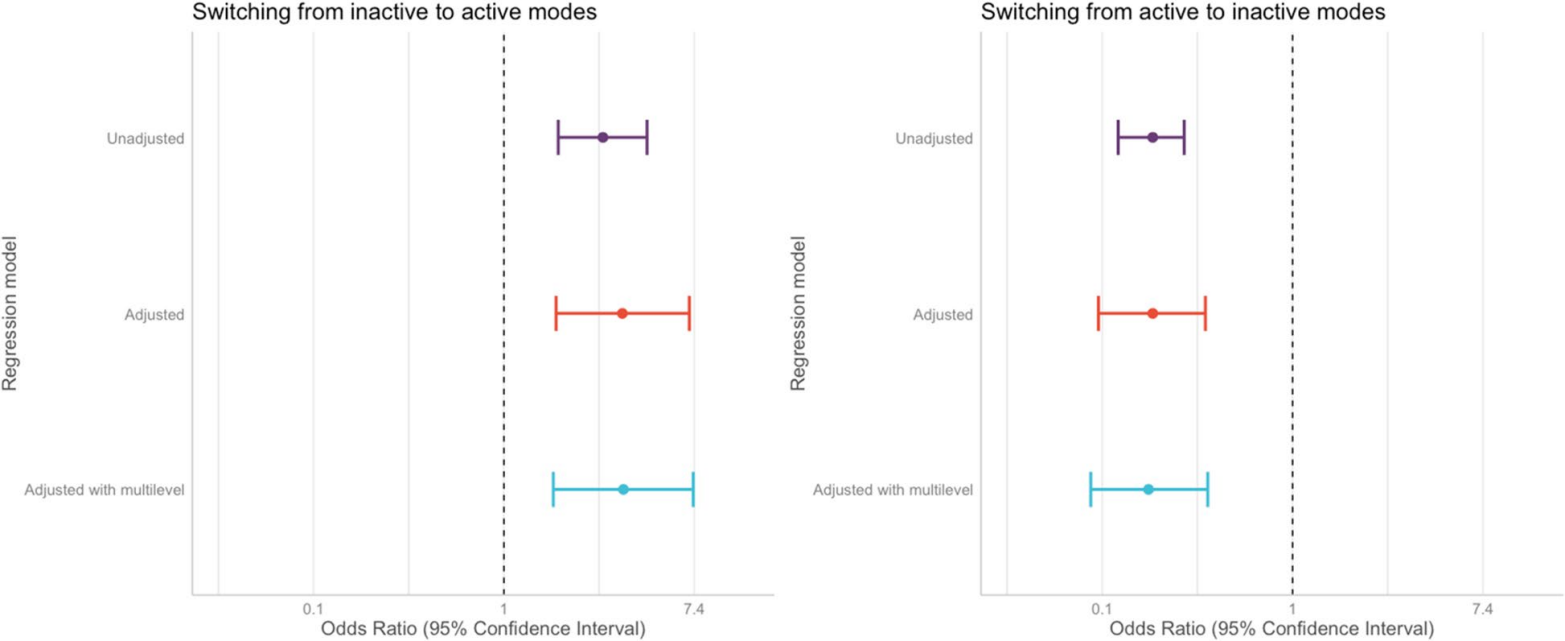
Regression model results from unadjusted, adjusted, and adjusted multilevel binomial logistic regression models Note: Adjusted and adjusted multilevel models are adjusted for by child age, sex, ethnicity, parent’s employment and occupation status, distance to school, household car ownership, and neighbourhood deprivation and crime quintile. In addition, multilevel models include clustering based on the child’s school

The intervention group was more likely (Odds Ratio [OR] 2.83; 95% Confidence Interval [CI] 1.77–4.50) to shift from inactive to active modes of travel for travel to school ‘today’ than those who were in the non-intervention group (Fig. 3 and Table 3). There may have been evidence of negative confounding, as the effect size of the fully adjusted model (OR 3.47; 95% CI 1.73–7.02) and the fully adjusted multilevel model (OR 3.51; 95% CI 1.68–7.31) were greater than that of the crude model. Similarly, children in the intervention group were less likely (unadjusted OR 0.22, 95% CI 0.15–0.31) to switch from active to inactive modes than the non-intervention group. The effect sizes of the fully adjusted (OR 0.23, 95% CI 0.13–0.40) and multilevel model (OR 0.22, 0.12–0.41)(OR 0.14, 95% CI 0.08–0.23) and multilevel model (OR 0.11, 0.05–0.24) were smaller similar tothan that of the crude model. Sensitivity analyses showed that the findings were similar when modelling usual travel mode to school and with a recategorised inactive mode outcome (Appendix Table 5 and 6).

**Table 3:** Unadjusted, adjusted, and adjusted multilevel binomial logistic regression models for odds of switching from inactive to active modes and switching from active to inactive modes ‘today’

	Inactive to Active			Active to Inactive		
Predictor variable	Unadjusted model	Adjusted model	Adjusted multilevel model	Unadjusted model	Adjusted model	Adjusted multilevel model
	OR	OR	OR	OR	OR	OR
	(95% CI)	(95% CI)	(95% CI)	(95% CI)	(95% CI)	(95% CI)
Constant	0.26	**0.38**	**0.33**	0.26	**0.05**	**0.05**
	(0.20 - 0.33)	**(0.02 - 7.98)**	**(0.01 - 7.97)**	(0.21 - 0.32)	**(0.00 - 0.73)**	**(0.00 - 0.80)**
London	2.83	**3.47**	**3.51**	0.23	**0.23**	**0.22**
	(1.77 - 4.50)	**(1.73 - 7.02)**	**(1.68 - 7.31)**	(0.16 - 0.32)	**(0.13 - 0.40)**	**(0.12 - 0.41)**
Sex (Female)		**1.11**	**1.11**		**0.86**	**0.85**
*Ref: Male*		**(0.63 - 1.97)**	**(0.63 - 1.98)**		**(0.55 - 1.35)**	**(0.54 - 1.34)**
Age		**1.05**	**1.06**		**1.03**	**1.03**
		**(0.71 - 1.53)**	**(0.71 - 1.58)**		**(0.76 - 1.40)**	**(0.75 - 1.42)**
Ethnicity (White)		**2.18**	**2.19**		**0.69**	**0.67**
*Ref: BAME*		**(1.18 - 4.07)**	**(1.17 - 4.12)**		**(0.42 - 1.13)**	**(0.40 - 1.12)**
Distance to school (Near ≤0.86 km)		**3.23**	**3.32**		**0.27**	**0.26**
*Ref: Far (>0.86 km)*		**(1.75 - 6.03)**	**(1.75 - 6.30)**		**(0.16 - 0.42)**	**(0.16 - 0.42)**
Vehicle ownership (Yes)		**0.13**	**0.13**		**16.47**	**17.18**
*Ref: No*		**(0.05 - 0.33)**	**(0.05 - 0.34)**		**(5.85 - 69.06)**	**(5.17 - 57.09)**
Employment (Part - time)		**1.23**	**1.22**		**1.30**	**1.28**
*Ref: Full - time*		**(0.61 - 2.45)**	**(0.60 - 2.47)**		**(0.73 - 2.30)**	**(0.72 - 2.28)**
Employment (Unemployed)		**1.02**	**1.02**		**1.14**	**1.13**
*Ref: Full - time*		**(0.46 - 2.19)**	**(0.46 - 2.24)**		**(0.63 - 2.06)**	**(0.61 - 2.06)**
Employment (Other)		**1.56**	**1.60**		**0.68**	**0.67**
*Ref: Full - time*		**(0.57 - 4.09)**	**(0.59 - 4.37)**		**(0.30 - 1.49)**	**(0.30 - 1.51)**
IDACI quintile (linear)		**0.76**	**0.72**		**1.68**	**1.72**
		**(0.18 - 2.67)**	**(0.19 - 2.77)**		**(0.82 - 3.33)**	**(0.84 - 3.51)**
Crime quintile (linear)		**0.61**	**0.64**		**1.84**	**1.91**
		**(0.15 - 1.96)**	**(0.18 - 2.28)**		**(0.85 - 3.91)**	**(0.86 - 4.26)**
Observations	469	**331**	**331**	1459	**985**	**985**
R^2^	0.043	**0.206**	**0.296**	0.053	**0.167**	**0.519**
ICC			**0.03**			**0.03**

*OR* Odds ratio, *95% CI* 95% Confidence interval, *ICC* Intraclass correlation coefficient

Table 4 shows the results for interaction effects between the intervention group and whether effects on whether children switched travel modes depend on the child’s sex, age, ethnicity, distance to school and vehicle ownership. Distance to school was the only variable that approached statistical significance in moderating the intervention's effect on switching from inactive to active modes of transport. Specifically, the interaction coefficient was OR 0.30 (95% CI 0.08–1.14), indicating that the intervention's impact varied depending on the distance to school. Stratified analyses revealed that among children living further from school (> 0.86 km), those in London were significantly more likely to switch to active modes of transport compared to children in Luton (OR 5.20; 95% CI 1.67–15.21). Conversely, among children living closer to school, there was no significant evidence of an intervention effect (OR 1.54; 95% CI 0.33–7.21).

**Table 4:** Adjusted multilevel logistic regression models with interaction terms

	**Switching from Inactive to Active modes**					**Switching from Active to Inactive modes**				
	**Model 1**	**Model 2**	**Model 3**	**Model 4**	**Model 5**	**Model 1**	**Model 2**	**Model 3**	**Model 4**	**Model 5**
Predictor variable	OR	OR	OR	OR	OR	OR	OR	OR	OR	OR
	(95% CI)	(95% CI)	(95% CI)	(95% CI)	(95% CI)	(95% CI)	(95% CI)	(95% CI)	(95% CI)	(95% CI)
Constant	0.35	0.11	0.36	0.31	0.24	0.05	0.14	0.05	0.05	0.11
	(0.01 - 8.37)	(0.00 - 5.99)	(0.01 - 8.88)	(0.01 - 7.26)	(0.01 - 5.90)	(0.00 - 0.81)	(0.00 - 5.58)	(0.00 - 0.78)	(0.00 - 0.79)	(0.01 - 2.22)
Site (London) *Ref: Luton*	3.18	84.84	3.1	5.2	17.4	0.22	0.02	0.32	0.24	0.05
	(1.18 - 8.58)	(0.10 - 99.63)	(1.34 - 7.18)	(2.23 - 12.10)	(1.56 - 19.16)	(0.10 - 0.49)	(0.00 - 3.60)	(0.15 - 0.65)	(0.12 - 0.50)	(0.00 - 0.57)
Sex (Female) *Ref: Male*	1.05	1.12	1.12	1.06	1.12	0.84	0.85	0.85	0.84	0.85
	(0.52 - 2.11)	(0.63 - 2.00)	(0.63 - 1.99)	(0.60 - 1.89)	(0.63 - 2.00)	(0.48 - 1.49)	(0.54 - 1.34)	(0.54 - 1.35)	(0.53 - 1.33)	(0.54 - 1.35)
Age	1.06	1.23	1.06	1.06	1.04	1.03	0.9	1.01	1.03	1.03
	(0.71 - 1.58)	(0.74 - 2.04)	(0.71 - 1.58)	(0.71 - 1.58)	(0.70 - 1.55)	(0.75 - 1.42)	(0.58 - 1.40)	(0.74 - 1.38)	(0.75 - 1.42)	(0.75 - 1.41)
Ethnicity (White) *Ref: BAME*	2.2	2.22	1.96	2.2	2.24	0.67	0.68	0.92	0.67	0.68
	(1.17 - 4.12)	(1.17 - 4.21)	(0.95 - 4.07)	(1.18 - 4.12)	(1.20 - 4.20)	(0.40 - 1.12)	(0.41 - 1.15)	(0.49 - 1.75)	(0.40 - 1.12)	(0.41 - 1.13)
Distance to school (Near ≤0.86 km)	3.33	3.42	3.33	4.76	3.27	0.26	0.26	0.26	0.28	0.26
*Ref: Far (>0.86 km)*	(1.76 - 6.33)	(1.78 - 6.55)	(1.76 - 6.33)	(2.23 - 10.13)	(1.73 - 6.18)	(0.16 - 0.42)	(0.16 - 0.42)	(0.16 - 0.42)	(0.15 - 0.50)	(0.16 - 0.43)
Vehicle ownership (Yes) *Ref: No*	0.13	0.12	0.13	0.12	0.22	17.19	17.08	16.91	17.33	7.3
	(0.05 - 0.34)	(0.05 - 0.33)	(0.05 - 0.34)	(0.05 - 0.33)	(0.07 - 0.73)	(5.17 - 57.13)	(5.14 - 56.74)	(5.09 - 56.11)	(5.21 - 57.63)	(1.57 - 33.9)
London * Sex (Female)	1.20					1.01				
	(0.34 - 4.21)					(0.39 - 2.60)				
		0.67					1.33			
		(0.29 - 1.56)					(0.70 - 2.53)			
			1.51					0.42		
			(0.38 - 6.03)					(0.14 - 1.25)		
				0.30					0.30	
				(0.08 - 1.14)					(0.09 - 1.00)	
					0.17					1.24
					(0.01 - 2.01)					(0.00 - 5.36)
R^2^	0.295	0.309	0.293	0.307	0.312	0.519	0.518	0.52	0.657	0.647
ICC	0.03	0.04	0.03	0.01	0.02	0.03	0.03	0.02	0.22	0.21

Model 1: Multilevel model with an interaction term for sex adjusted by age, ethnicity, distance to school, vehicle ownership, parents’ employment and occupation status, neighbourhood deprivation and crime quintile; Model 2: Multilevel model with an interaction term for age adjusted by gender, ethnicity, distance to school, vehicle ownership, parents’ employment and occupation status, neighbourhood deprivation and crime quintile; Model 3: Multilevel model with an interaction term for ethnicity adjusted by gender, age, distance to school, vehicle ownership, parents’ employment and occupation status, neighbourhood deprivation and crime quintile; Model 4: Multilevel model with an interaction term for distance to school adjusted by gender, age, ethnicity, vehicle ownership, parents’ employment and occupation status, neighbourhood deprivation and crime quintile; Model 5: Multilevel model with an interaction term for vehicle ownership adjusted by gender, age, ethnicity, distance to school, parents’ employment and occupation status, neighbourhood deprivation and crime quintile

*OR* Odds ratio, *CI* Confidence interval, *ICC* Intraclass correlation coefficient

## Discussion

This study showed that the implementation of London’s ULEZ in April 2019 resulted in positive modal shifts in children’s travel to school. Over a one-year study period, we found that children attending schools within the ULEZ were more likely to switch to active travel modes, and less likely to switch to inactive travel modes than children in the comparison group in Luton. The impact on switching to active travel was most pronounced in those living further away from school.

Post-ULEZ implementation in Central London, there was a drop of up to 9% in total vehicle counts and 34% in non-compliant vehicle counts, with no clear evidence of traffic displacement to nearby areas [30]. This suggests ULEZ effectively curbed non-compliant vehicle journeys, possibly encouraging a shift to active or public transport. This was also seen in a study examining Madrid's CAZ which demonstrated that private vehicle use decreased, and active travel and public transport use increased post implementation [16]. However, neither of these assessments examined changes in transport modes using formal statistical analyses, nor did they include a control group, making it difficult to attribute the observed changes solely to the CAZ. Moreover, the study assessing the Madrid CAZ was cross-sectional and causal relationships could not be inferred [16].

Our study revealed a significant interaction between ULEZ implementation, school distance, and the odds of shifting from inactive to active travel. Specifically, children living further from school in London were more likely to make this shift, with no effect observed in those living closer to school. Children living closer to school may have switched transport modes regardless of whether the ULEZ was implemented. Previous studies have highlighted the crucial role of school distance in choosing active transport, with car travel in London and urban Spain among children of a similar age group (aged 9–10) being more likely when the home-school distance exceeded 0.50 miles (0.80 km) and 0.54 miles (0.88 km), respectively [26, 27].

### Strengths and weaknesses

This study's strengths include its prospective design, large sample size, its ethnically and socioeconomically diverse study population, and use of longitudinal data, which enhances causal inference. The inclusion of a comparison group and control for potential confounders increase confidence that observed changes resulted from the intervention. By accounting for the hierarchical nature of the data using multilevel modelling, we could adjust standard error estimates for the impact of clustering at the school level. Moreover, the year-long interval between baseline and follow-up data accounted for seasonal variations potentially affecting travel mode choice.

Limitations of this study include the potential for social desirability and recall bias in self-reported travel modes, though this was mitigated by asking children their transport method on the day of assessment. While such self-reporting has shown validity and reliability among US children aged 8–11, it may not be generalizable to other contexts with varying travel options [22]. Although we use a single day measure as our outcome measure, which may not represent travel modes on other days of the week, findings from our sensitivity analyses examining usual travel to school were consistent with those in our main analysis using travel to school ‘today’. Moreover, travel options may vary between morning and afternoon school commutes, due to greater time constraints in the morning. Previous UK-based research has however shown a high correlation between travel mode to and from school [31]. Our study's focus on travel to school may nevertheless underestimate the impact; future studies on the ULEZ policy's impact on children's travel might benefit from including data on both travel to and from school [32, 33]. In addition, the outcome measure of a modal shift does not necessarily represent a change in physical activity levels, which is a more proximal measure that can affect health outcomes. However, a longitudinal study of British children aged 9–10 years children found a significant positive association between children who changed their mode of travel to school and minutes of daily moderate-to-vigorous physical activity [34].

Although our study adjusted for differences between the London and Luton cohorts based on a range of demographic variables, other unmeasured confounders may have impacted transport mode choice. For instance, there may be differences in transport contexts, including the scale and quality of pedestrian, cycling, and public transport infrastructure. In addition, other policies that may have been introduced during the study period, such as low traffic neighbourhoods (LTNs) and School Street schemes aimed at reducing access or convenience for motorised vehicles, could have impacted decisions to switch to active travel. These schemes, however, were largely introduced during Covid-19, or after the study period, and would thus have a limited impact. Future natural experimental evaluations should seek to include multiple control groups matched on variables that are likely to be important sources of bias, as recommended by UK Medical Research Council’s guidance [35].

In addition, there is a need to examine changes to modal shifts across more time points to determine whether modal shifts persist. This analysis was initially designed to measure changes in active travel behaviour across four years, however this aim was truncated due to Covid-19 restrictions to a more limited consideration of the data collected in the years pre- and post-implementation of the ULEZ. The Covid-19 restrictions also meant that data could not be collected from a few schools in either site, specifically those that were due for assessment between mid-March to July 2020, meaning some data were systematically missing (i.e., not at random). Thus, we did not perform multiple imputation, which would have preserved sample size and statistical power, as it is not recommended when missing data is not random [36]. Therefore, the children included in this study may not be wholly representative of the target population, which may have biased results. Moreover, the composition of the study cohort along with implementation of multiple overlapping strategies in London to reduce traffic emissions, makes it challenging to attribute changes specifically to the ULEZ, as opposed to a broader range of policies. Thus, it is not easy to simplistically apply lessons learnt from the ULEZ to other CAZs, without careful consideration of broader context of regional and national air quality policies.

### Policy implications and recommendations for future research

We found that the introduction of the Central London ULEZ was associated with shifting children’s transport to school from inactive to active modes, suggesting that vehicle restriction schemes using financial disincentives may play an important role in promoting shifts towards active travel. Scaling up current policies (such as the 2021 ULEZ expansion), or introducing similar policies in other cities may therefore help the UK government achieve its target of increasing the share of children walking to school from 49% in 2014 to 55% by 2025 [37], as well as the Mayor of London’s target of 60% of children walking to school by 2026 [38]. Changing the way children travel to school can have significant effects on congestion, air pollution emissions, and levels of physical activity, as about a quarter of car trips during peak morning hours in London are made for school drop-offs [39].

A small number of studies have assessed interventions employing solely negative motivators (e.g., vehicle restrictions, financial disincentives), which may more effectively alter driving behavior compared to positive strategies [15]. Most research on promoting active travel in children focuses on positive strategies such as walking school buses, cycling training, infrastructure improvements, campaigns, and incentives [40, 41]. Further rigorous evaluations of vehicle restriction policies' health impacts on children are necessary [42]. With more CAZs, LTNs, and School Streets being implemented, such studies will become increasingly feasible.

Future analyses should consider the impact of such policies on active travel behaviours and the potential of this to deliver additional health benefits to children. Understanding which processes trigger such changes, be it increased driving costs, perceived safety improvements, or reduced pollution, can help design policies that optimize emission reduction and active travel promotion.

## Conclusion

We found that children attending schools within the ULEZ area were more likely to switch from inactive to active travel modes, and that this change was greatest among children who lived furthest from their school. Children in the London cohort were also less likely to switch from active to inactive modes. These results underline the dual benefits of vehicle restriction policies for reducing pollution and promoting active travel among primary school aged children in London. Future analyses of vehicle restriction policies should incorporate a consideration of their impact on active travel behaviours and the potential of this to deliver additional health benefits to children. In addition, further investigation into the processes that contribute to transport behavioural change are warranted. The ULEZ expanded to all London boroughs in August 2023 [43], which may be a further opportunity to increase children’s active travel to school and promote children’s health and wellbeing.

## Supplementary Information

Supplementary Material 1

## Data Availability

The data that support the findings of this study are available from the Children’s Health in London and Luton (CHILL) study (https://www.qmul.ac.uk/chill/) but restrictions apply to the availability of these data (analyses are continuing) and so are not publicly available. Interested individuals can apply to the CHILL databank for access, and once approved, can apply to the corresponding author.

## Abbreviations

UK: United Kingdom
CAZ: Clean air zones
NO_x_: Nitrous oxides
PM: Particulate matter
CO_2_: Carbon dioxide
ULEZ: Ultra low emission zone
CHILL: Children’s Health in London and Luton
SD: Standard deviation
IDACI: Income Deprivation Affecting Children Index
BAME: Black, Asian, and Minority Ethnic
OR: Odds ratio
CI: Confidence interval
US: United States
LTNs: Low traffic neighbourhoods
