# Correction: Children’s Health in London and Luton (CHILL) cohort: a 12-month natural experimental study of the effects of the Ultra Low Emission Zone on children’s travel to school

**DOI:** 10.1186/s12966-025-01791-y

**Published:** 2025-08-05

**Authors:** Christina Xiao, James Scales, Jasmine Chavda, Rosamund E. Dove, Ivelina Tsocheva, Helen E. Wood, Harpal Kalsi, Luke Sartori, Grainne Colligan, Jessica Moon, Esther Lie, Kristian Petrovic, Bill Day, Cheryll Howett, Amanda Keighley, Borislava Mihaylova, Veronica Tofolutti, Jonathan Grigg, Gurch Randhawa, Aziz Sheikh, Monica Fletcher, Ian Mudway, Sean Beevers, W. James Gauderman, Christopher J. Grifths, Esther van Sluijs, Jenna Panter

**Affiliations:** 1 MRC Epidemiology Unit, School of Clinical Medicine, University of Cambridge, Box 285, Cambridge, UK; 2 Centre for Primary Care, Wolfson Institute of Population Health, Barts and the London School of Medicine and Dentistry, Queen Mary University of London, London, UK; 3 Asthma UK Centre for Applied Research, Edinburgh, UK; 4 Institute for Health Research, University of Bedfordshire, Luton, UK; 5 Social Action for Health, London, UK; 6 Centre of the Cell, Queen Mary University of London, London, UK; 7 Health Economics and Policy Research Unit, Wolfson Institute of Population Health, Queen Mary University of London, London, UK; 8 Nufeld Department of Population Health, University of Oxford, Oxford, UK; 9 Blizard Institute, Faculty of Medicine and Dentistry, Queen Mary University of London, London, UK; 10 Usher Institute, University of Edinburgh, Edinburgh, UK; 11 MRC Asthma UK Centre in Allergic Mechanisms of Asthma, London, UK; 12 MRC Centre for Environment and Health, Imperial College London, London, UK; 13 and Chemical and Radiation Threats and Hazards, NIHR Health Protection Research Units in Environmental Exposures and Health, Imperial College London, London, UK; 14 Keck School of Medicine, University of Southern California, Los Angeles, USA

**Correction** **:** **Xiao et al. Int J Behav Nutr Phys Act 21, 89 (2024)**

**https://doi.org/10.1186/s12966-024-01621-7**

Following publication of the original article, the authors identified coding errors in the merging of datasets. Correcting these has resulted in an increased number of participants with complete covariate data. These corrections have had a minimal impact on the main findings; the magnitude of the effects and the primary conclusions remain supported.

**Table:** 

Page (PDF)	Incorrect	Correct
1	…Among children who took inactive modes at baseline, 42% of children in London and 20% of children in Luton switched to active modes. For children taking active modes at baseline, 5% of children in London and 21% of children in Luton switched to inactive modes. Relative to the children in Luton, children in London were more likely to have switched from inactive to active modes (OR 3.64, 95% CI 1.21–10.92). Children in the intervention group were also less likely to switch from active to inactive modes (OR 0.11, 0.05–0.24). Moderator analyses showed that children living further from school were more likely to switch from inactive to active modes (OR 6.06,1.87–19.68) compared to those living closer (OR 1.43, 0.27–7.54)	…Among children who took inactive modes at baseline, 42% of children in London and 20% of children in Luton switched to active modes. For children taking active modes at baseline, 5% of children in London and 21% of children in Luton switched to inactive modes. Relative to the children in Luton, children in London were more likely to have switched from inactive to active modes (**OR 3.51, 95% CI 1.68–7.31**). Children in the intervention group were also less likely to switch from active to inactive modes (**OR 0.22, 0.12–0.41**). Moderator analyses showed that children living further from school were more likely to switch from inactive to active modes **(OR 5.20; 1.67–15.21**) compared to those living closer (**OR 1.54, 0.33–7.21**)
	This value was transformed into a binary variable using a 0.78-km cut-off,	This value was transformed into a binary variable using a **0.86-km** cut-off,
5	…distance to school (≤ 0.78 km or > 0.78 km),	…distance to school (≤ 0.86 km or > 0.86 km),
		*A new paragraph needs to be inserted between the 1 st and 2nd paragraph of Data Analysis, as follows:* We encountered convergence issues in the fully adjusted multilevel model for switching from active to inactive modes, likely due to overparameterization. To resolve this, we removed the'parental occupation'variable, as socioeconomic factors were already captured by other covariates (parental employment, crime, IDACI). Sensitivity analyses showed that this exclusion did not significantly affect model fit (likelihood ratio test *p* = 0.964) and improved AIC and BIC values (Appendix Table 3), indicating a better-fitting, more parsimonious
	We conducted two sensitivity analyses	We conducted additional sensitivity analyses
	Children who were not included in the analysis at any time point (reasons for exclusion can be found in Fig. 2) from the London cohort (*n* = 664) were more likely to be male (48.5% vs. 42.4%), from a minority ethnic background (70.1% vs. 66.3%) and lived closer to school (52.7% vs. 48.0%) compared to children living in London who were included in the analyses (Appendix Table 3). Luton children not included in the analyses (*n* = 768) were more likely to be older (7.9 vs. 7.7 years) at baseline, less likely to have parents in full-time employment (28.9% vs. 34.9%), more likely to live further from school (63.1% vs. 54.4%) and lived in areas with lower levels of neighbourhood deprivation than children living in Luton who were included in the analyses	Children who were not included in the analysis at any time point (reasons for exclusion can be found in Fig. 2) from the London cohort (*n* = 664) were more likely to be male (48.5% vs. 42.4%), from a minority ethnic background (70.1% vs. 66.3%), more likely to have an ‘Other’ occupation (26.8% vs. 18.7%), and lived in areas with higher crime and neighbourhood deprivation compared to children living in London who were included in the analyses (Appendix Table 4). Luton children not included in the analyses (*n* = 768) were more likely to be older (7.9 vs. 7.7 years) at baseline, less likely to have parents in full-time employment (28.9% vs. 34.9%), more likely to live further from (58.9% vs. 51.7%) and lived in areas with higher levels of crime and neighbourhood deprivation than children living in Luton who were included in the analyses
6	There may have been evidence of negative confounding, as the effect size of the fully adjusted model (OR 3.02; 95% CI 1.60–5.70) and the fully adjusted multilevel model (OR 3.64; 95% CI 1.21–10.92) were greater than that of the crude model	There may have been evidence of negative confounding, as the effect size of the fully adjusted model (OR 3.47; 95% CI 1.73–7.02) and the fully adjusted multilevel model (OR 3.51; 95% CI 1.68–7.31) were greater than that of the crude model
	The effect sizes of the fully adjusted (OR 0.14, 95% CI 0.08–0.23) and multilevel model (OR 0.11, 0.05–0.24) were smaller than that of the crude model	The effect sizes of the fully adjusted (OR 0.23, 95% CI 0.13–0.40) and multilevel model (OR 0.22, 0.12–0.41) were smaller similar to than that of the crude model
	Appendix Table 4 and 5	Appendix Table 5 and 6
7	Only distance to school statistically significantly moderated the intervention’s effect on switching from inactive to active modes of transport. Specifically, the interaction coefficient was OR 0.24 (95% CI 0.06– 0.88), indicating that the intervention’s impact varied depending on the distance to school. Stratified analyses revealed that among children living further from school (> 0.78 km), those in London were significantly more likely to switch to active modes of transport compared to children in Luton (OR 6.06; 95% CI 1.87–19.68). Conversely, among children living closer to school, there was no significant evidence of an intervention effect (OR 1.43; 95% CI 0.27–7.54)	Distance to school was the only variable that approached statistical significance in moderating the intervention's effect on switching from inactive to active modes of transport. Specifically, the interaction coefficient was OR 0.30 (95% CI 0.08–1.14), indicating that the intervention's impact varied depending on the distance to school. Stratified analyses revealed that among children living further from school (> 0.86 km), those in London were significantly more likely to switch to active modes of transport compared to children in Luton (OR 5.20; 95% CI 1.67–15.21). Conversely, among children living closer to school, there was no significant evidence of an intervention effect (OR 1.54; 95% CI 0.33–7.21)

Moreover, all tables, Fig. 3 and Supplementary Materials need to be updated, as follows:

**Table 1:** Descriptive baseline characteristics of the study population*

Covariate	London (*n* = 1000)		Luton (*n* = 982)		*p*-value*
Age (mean, SD)					
Baseline	7.9	(0.9)	7.7	(0.9)	< 0.001
Follow-up	8.9	(0.9)	8.7	(0.7)	< 0.001
Sex (n, %)					
Male	424	(42.4)	490	(49.9)	0.002
Female	576	(57.6)	492	(50.1)	
Ethnicity (n, %)					
BAME	629	(66.3)	572	(59.8)	< 0.001
White	320	(33.7)	384	(40.2)	
Employment status (n, %)					
Full-time	279	(32.2)	317	(34.9)	0.038
Part-time	224	(25.9)	231	(25.4)	
Unemployed	126	(14.5)	119	(13.1)	
Other	237	(27.4)	241	(26.5)	
Occupational category (n, %)					
Professional/Managerial	368	(56.0)	310	(45.7)	0.007
Skilled	96	(14.6)	112	(16.5)	
Unskilled	70	(10.7)	79	(11.7)	
Other	123	(18.7)	177	(26.1)	
Distance to school (n, %)					
Near (≤ 0.86 km)	**475**	**(51.3)**	**351**	**(48.3)**	**0.254**
Far (> 0.86 km)	**451**	**(48.7)**	**375**	**(51.7)**	
Vehicle ownership** (n, %)					
Yes	461	(54.1)	798	(89.7)	< 0.001
No	391	(45.9)	92	(10.3)	
Crime Quintile (n, %)					
1 (highest crime)	**309**	**(31.0)**	**302**	**(30.9)**	< 0.001
2	**301**	**(30.2)**	**328**	**(33.6)**	
3	**172**	**(17.2)**	**237**	**(24.3)**	
4	**115**	**(11.5)**	**89**	**(9.1)**	
5 (lowest crime)	**101**	**(10.1)**	**21**	**(2.1)**	
IDACI Quintile (n, %)					
1 (highest level of deprivation)	**578**	**(57.9)**	**190**	**(19.4)**	< 0.001
2	**269**	**(27.0)**	**368**	**(37.7)**	
3	**75**	**(7.5)**	**282**	**(28.9)**	
4	**34**	**(3.4)**	**114**	**(11.7)**	
5 (lowest level of deprivation)	**42**	**(4.2)**	**23**	**(2.4)**	

Sums of the number of participants with each characteristic may equal the total number of participants if data is missing

*N* Number, *BAME* Black, Asian, and Minority Ethnic, *SD* Standard deviation, *km* Kilometre, *IDACI* Index Deprivation Affecting Children Index

*p value refers to independent samples t-tests for continuous variables or Pearson's χ2 tests for categorical variables

**Vehicle ownership data was only collected at follow-up

**Table 2:** Proportion of children maintaining or switching modes in London and Luton

Baseline	Follow-up	Group	London	Luton
			n (%)	n (%)
Active	Active	Maintained active modes	812 (94%)	475 (79%)
	Inactive	Switched to inactive modes	48 (6%)	124 (21%)
Inactive	Active	Switched to active modes	44 (42%)	74 (20%)
	Inactive	Maintained inactive modes	61 (58%)	290 (80%)

**Table 3:** Unadjusted, adjusted, and adjusted multilevel binomial logistic regression models for odds of switching from inactive to active modes and switching from active to inactive modes ‘today’

	Inactive to Active			Active to Inactive		
Predictor variable	Unadjusted model	Adjusted model	Adjusted multilevel model	Unadjusted model	Adjusted model	Adjusted multilevel model
	OR	OR	OR	OR	OR	OR
	(95% CI)	(95% CI)	(95% CI)	(95% CI)	(95% CI)	(95% CI)
Constant	0.26	**0.38**	**0.33**	0.26	**0.05**	**0.05**
	(0.20—0.33)	**(0.02—7.98)**	**(0.01—7.97)**	(0.21—0.32)	**(0.00—0.73)**	**(0.00—0.80)**
London	2.83	**3.47**	**3.51**	0.23	**0.23**	**0.22**
	(1.77—4.50)	**(1.73—7.02)**	**(1.68—7.31)**	(0.16—0.32)	**(0.13—0.40)**	**(0.12—0.41)**
Sex (Female)		**1.11**	**1.11**		**0.86**	**0.85**
*Ref: Male*		**(0.63—1.97)**	**(0.63—1.98)**		**(0.55—1.35)**	**(0.54—1.34)**
Age		**1.05**	**1.06**		**1.03**	**1.03**
		**(0.71—1.53)**	**(0.71—1.58)**		**(0.76—1.40)**	**(0.75—1.42)**
Ethnicity (White)		**2.18**	**2.19**		**0.69**	**0.67**
*Ref: BAME*		**(1.18—4.07)**	**(1.17—4.12)**		**(0.42—1.13)**	**(0.40—1.12)**
Distance to school (Near ≤ 0.86 km)		**3.23**	**3.32**		**0.27**	**0.26**
*Ref: Far (*> *0.86 km)*		**(1.75—6.03)**	**(1.75—6.30)**		**(0.16—0.42)**	**(0.16—0.42)**
Vehicle ownership (Yes)		**0.13**	**0.13**		**16.47**	**17.18**
*Ref: No*		**(0.05—0.33)**	**(0.05—0.34)**		**(5.85—69.06)**	**(5.17—57.09)**
Employment (Part—time)		**1.23**	**1.22**		**1.30**	**1.28**
*Ref: Full—time*		**(0.61—2.45)**	**(0.60—2.47)**		**(0.73—2.30)**	**(0.72—2.28)**
Employment (Unemployed)		**1.02**	**1.02**		**1.14**	**1.13**
*Ref: Full—time*		**(0.46—2.19)**	**(0.46—2.24)**		**(0.63—2.06)**	**(0.61—2.06)**
Employment (Other)		**1.56**	**1.60**		**0.68**	**0.67**
*Ref: Full—time*		**(0.57—4.09)**	**(0.59—4.37)**		**(0.30—1.49)**	**(0.30—1.51)**
IDACI quintile (linear)		**0.76**	**0.72**		**1.68**	**1.72**
		**(0.18—2.67)**	**(0.19—2.77)**		**(0.82—3.33)**	**(0.84—3.51)**
Crime quintile (linear)		**0.61**	**0.64**		**1.84**	**1.91**
		**(0.15—1.96)**	**(0.18—2.28)**		**(0.85—3.91)**	**(0.86—4.26)**
Observations	469	**331**	**331**	1459	**985**	**985**
R^2^	0.043	**0.206**	**0.296**	0.053	**0.167**	**0.519**
ICC			**0.03**			**0.03**

*OR* Odds ratio, *95% CI* 95% Confidence interval, *ICC* Intraclass correlation coefficient

**Table 4:** Adjusted multilevel logistic regression models with interaction terms

	**Switching from Inactive to Active modes**					**Switching from Active to Inactive modes**				
	**Model 1**	**Model 2**	**Model 3**	**Model 4**	**Model 5**	**Model 1**	**Model 2**	**Model 3**	**Model 4**	**Model 5**
Predictor variable	OR	OR	OR	OR	OR	OR	OR	OR	OR	OR
	(95% CI)	(95% CI)	(95% CI)	(95% CI)	(95% CI)	(95% CI)	(95% CI)	(95% CI)	(95% CI)	(95% CI)
Constant	0.35	0.11	0.36	0.31	0.24	0.05	0.14	0.05	0.05	0.11
	(0.01—8.37)	(0.00—5.99)	(0.01—8.88)	(0.01—7.26)	(0.01—5.90)	(0.00—0.81)	(0.00—5.58)	(0.00—0.78)	(0.00—0.79)	(0.01—2.22)
Site (London) *Ref: Luton*	3.18	84.84	3.1	5.2	17.4	0.22	0.02	0.32	0.24	0.05
	(1.18—8.58)	(0.10—99.63)	(1.34—7.18)	(2.23—12.10)	(1.56—19.16)	(0.10—0.49)	(0.00—3.60)	(0.15—0.65)	(0.12—0.50)	(0.00—0.57)
Sex (Female) *Ref: Male*	1.05	1.12	1.12	1.06	1.12	0.84	0.85	0.85	0.84	0.85
	(0.52—2.11)	(0.63—2.00)	(0.63—1.99)	(0.60—1.89)	(0.63—2.00)	(0.48—1.49)	(0.54—1.34)	(0.54—1.35)	(0.53—1.33)	(0.54—1.35)
Age	1.06	1.23	1.06	1.06	1.04	1.03	0.9	1.01	1.03	1.03
	(0.71—1.58)	(0.74—2.04)	(0.71—1.58)	(0.71—1.58)	(0.70—1.55)	(0.75—1.42)	(0.58—1.40)	(0.74—1.38)	(0.75—1.42)	(0.75—1.41)
Ethnicity (White) *Ref: BAME*	2.2	2.22	1.96	2.2	2.24	0.67	0.68	0.92	0.67	0.68
	(1.17—4.12)	(1.17—4.21)	(0.95—4.07)	(1.18—4.12)	(1.20—4.20)	(0.40—1.12)	(0.41—1.15)	(0.49—1.75)	(0.40—1.12)	(0.41—1.13)
Distance to school (Near ≤ 0.86 km)	3.33	3.42	3.33	4.76	3.27	0.26	0.26	0.26	0.28	0.26
*Ref: Far (*> *0.86 km)*	(1.76—6.33)	(1.78—6.55)	(1.76—6.33)	(2.23—10.13)	(1.73—6.18)	(0.16—0.42)	(0.16—0.42)	(0.16—0.42)	(0.15—0.50)	(0.16—0.43)
Vehicle ownership (Yes) *Ref: No*	0.13	0.12	0.13	0.12	0.22	17.19	17.08	16.91	17.33	7.3
	(0.05—0.34)	(0.05—0.33)	(0.05—0.34)	(0.05—0.33)	(0.07—0.73)	(5.17—57.13)	(5.14—56.74)	(5.09—56.11)	(5.21—57.63)	(1.57—33.9)
London * Sex (Female)	1.20					1.01				
	(0.34—4.21)					(0.39—2.60)				
		0.67					1.33			
		(0.29—1.56)					(0.70—2.53)			
			1.51					0.42		
			(0.38—6.03)					(0.14—1.25)		
				0.30					0.30	
				(0.08—1.14)					(0.09—1.00)	
					0.17					1.24
					(0.01—2.01)					(0.00—5.36)
R^2^	0.295	0.309	0.293	0.307	0.312	0.519	0.518	0.52	0.657	0.647
ICC	0.03	0.04	0.03	0.01	0.02	0.03	0.03	0.02	0.22	0.21

*OR* Odds ratio, *CI* Confidence interval, *ICC* Intraclass correlation coefficient

**Fig. 3:**
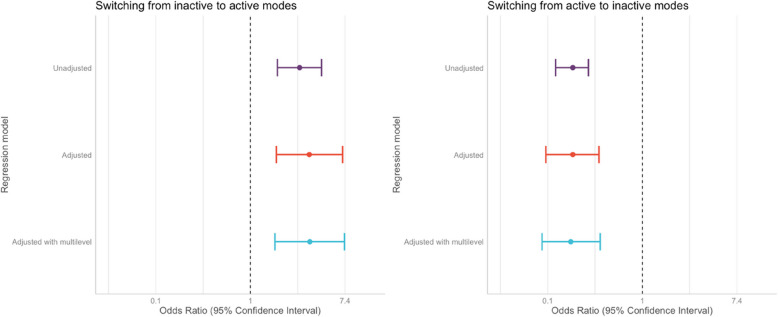
Regression model results from unadjusted, adjusted, and adjusted multilevel binomial logistic regression models. Note: Adjusted and adjusted multilevel models are adjusted for by child age, sex, ethnicity, parent’s employment and occupation status, distance to school, household car ownership, and neighbourhood deprivation and crime quintile. In addition, multilevel models include clustering based on the child’s school

The original article has been updated accordingly.

## Supplementary Information

Supplementary Material 1.

