# Supplementary material for: Children’s Health in London and Luton (CHILL) cohort: a 12-month natural experimental study of the effects of the Ultra Low Emission Zone on children’s travel to school
Source: Int J Behav Nutr Phys Act. 2024 Sep 5;21:89. doi: 10.1186/s12966-024-01621-7 (PMC11375866; doi:10.1186/s12966-024-01621-7)
Supplement: Supplementary file 1 — Supplementary Material 1 [file 12966_2024_1621_MOESM1_ESM.docx]

# Supplementary Material

**Tables**

[**Appendix Table 1 Self-reported mode of transport to school questions on day of annual health assessment** 3](#_Toc195458664)

[**Appendix Table 2 Questions used from parental surveys** 4](#_Toc195458665)

[**Appendix Table 3 Sensitivity analysis of including occupation within the model for participants switching from inactive to active modes** 6](#_Toc195458666)

[**Appendix Table 4 Descriptive characteristics of included and excluded participants in either London or Luton** 7](#_Toc195458667)

[**Appendix Table 5 Sensitivity analysis of unadjusted, adjusted, and adjusted multilevel binomial logistic regression models for odds of switching from inactive to active modes and switching from active to inactive modes ‘usually’** 9](#_Toc195458668)

[**Appendix Table 6 Sensitivity analysis of unadjusted, adjusted, and adjusted multilevel binomial logistic regression models for odds of switching from inactive to active modes and switching from active to inactive modes with re-categorised inactive transport mode variable** 10](#_Toc195458669)

**Figures**

[**Appendix Figure 1 Theoretical framework of how Clean Air Zones can impact children’s health** 2](#_Toc157455157)

**Appendix Figure 1 Theoretical framework of how Clean Air Zones can impact children’s health**

**Appendix Table 1 Self-reported mode of transport to school questions on day of annual health assessment**

| **Annual Health Assessment** | | **Creating new variables for analysis** |
| --- | --- | --- |
| How did you travel to school today? | Checkbox, required   \| 1 \| Walk \| \| --- \| --- \| \| 2 \| Scooter \| \| 3 \| Bike \| \| 4 \| Private car \| \| 5 \| Taxi \| \| 6 \| Bus \| \| 7 \| Train/Tube \| \| 8 \| Other \| \| 9999 \| Not answered \| | \| Active \| Walk  Scooter  Bike  Bus  Train/Tube \| \| --- \| --- \| \| Inactive \| Private car  Taxi \| \| NA \| Other  Not answered \| |
| How do you usually travel to school? | Checkbox, required   \| 1 \| Walk \| \| --- \| --- \| \| 2 \| Scooter \| \| 3 \| Bike \| \| 4 \| Private car \| \| 5 \| Taxi \| \| 6 \| Bus \| \| 7 \| Train/Tube \| \| 8 \| Other \| \| 9999 \| Not answered \|   . | \| Active \| Walk  Scooter  Bike  Bus  Train/Tube \| \| --- \| --- \| \| Inactive \| Private car  Taxi \| \| NA \| Other  Not answered \| |

**Appendix Table 2 Questions used from parental surveys**

| **Parental survey: About your child** | | **Variables used for analysis** |
| --- | --- | --- |
| Date of birth | DD-MMM-YYYY | Child age |
| Gender | Checkbox, required   \| 1 \| Male \| \| --- \| --- \| \| 2 \| Female \|   . | \| Child sex \| \| \| --- \| --- \| \| 1 \| Male \| \| 2 \| Female \| |
| Ethnicity | Checkbox, required   \| Asian or Asian British \| \| \| --- \| --- \| \| 1 \| Indian \| \| 2 \| Pakistani \| \| 3 \| Bangladeshi \| \| 4 \| Any other Asian background \| \| Black or Black British \| \| \| 5 \| Caribbean \| \| 6 \| African \| \| 7 \| Any other Black background \| \| Mixed \| \| \| 8 \| White and Black Caribbean \| \| 9 \| White and Black African \| \| 10 \| White and Asian \| \| 11 \| Any other mixed background \| \| White \| \| \| 12 \| British \| \| 13 \| Irish \| \| 14 \| Any other White background \| \| Chinese or any other ethnic group \| \| \| 15 \| Chinese \| \| 16 \| Any other ethnic group \|   . | \| Child ethnicity \| \| \| --- \| --- \| \| White \| British  Irish  Any other White background \| \| British, Asian, and Minority Ethnic \| Indian  Pakistani  Bangladeshi  Any other Asian background  Caribbean  African  Another other Black background  White and Black Caribbean  White and Black African  White and Asian  Any other mixed background  Chinese  Any other ethnic group \| |
| If other, please specify | text |  |
| Home address | House number/name and street  Town/City  Postcode |  |

| **Parental survey: About you** | |  |
| --- | --- | --- |
| Are you currently: | Dropdown, required   \| 1 \| Employed full-time \| \| --- \| --- \| \| 2 \| Employed part-time \| \| 3 \| Self-employed \| \| 4 \| Full-time parent/carer \| \| 5 \| Unemployed \| \| 6 \| Other \|   . | \| Parental employment status \| \| \| --- \| --- \| \| Full-time \| Employed full-time Employed part-time \| \| Part-time \| Employed part-time \| \| Unemployed \| Unemployed \| \| Other \| Self-employed  Other \| |
| If other, please specify | text |  |
| Which of these categories best describe your occupation? | Dropdown, required   \| 1 \| Professional \| \| --- \| --- \| \| 2 \| Managerial & technical \| \| 3 \| Skilled trade \| \| 4 \| Skilled non-manual \| \| 5 \| Skilled manual \| \| 6 \| Unskilled \| \| 7 \| Other/Unsure \|   . | \| Parental occupation \| \| \| --- \| --- \| \| Professional/  managerial \| Professional  Managerial & technical \| \| Skilled \| Skilled trade  Skilled non-manual  Skilled manual \| \| Unskilled \| Unskilled \| \| Other/Unsure \| Other \| |
| How many cars or vans are owned, or available for use, by members of your household?  Do not include motorcycles, scooters or mopeds.  Please write a number in the box, if none write '0' | Integer, Min: 0, Max: 9999, required | \| Car ownership \| \| \| --- \| --- \| \| $\geq$1 \| Yes \| \| <1 \| No \| |

**Appendix Table 3 Sensitivity analysis of including occupation within the model for participants switching from inactive to active modes**

|  | **Switching from inactive to active modes** | | | |
| --- | --- | --- | --- | --- |
|  | **Adjusted model** | | **Multilevel model** | |
| **Predictor variable** | **Without occupation** | **With occupation** | **Without occupation** | **With occupation** |
| AIC | 281 | 287 | 284 | 289 |
| BIC | 345 | 361 | 351 | 367 |
| Likelihood ratio test (p-value) |  | 0.821 |  | 0.820 |

|  | **London** | | | | | **Luton** | | | | |
| --- | --- | --- | --- | --- | --- | --- | --- | --- | --- | --- |
|  | **Included (n = 1000)** | | **Excluded (n= 664)** | | **p-value*** | **Included (n = 982)** | | **Excluded (n = 768)** | | **p-value*** |
|  |  |  |  |  |  |  |  |  |  |  |
| Baseline | 7.9 | (0.9) | 8.0 | (0.9) | 0.057 | 7.7 | (0.9) | 7.9 | (0.9) | <0.001 |
| Sex (n, %) |  |  |  |  |  |  |  |  |  |  |
| Male | 424 | (42.4) | 322 | (48.5) | 0.016 | 490 | (49.9) | 400 | (52.1) | 0.390 |
| Female | 576 | (57.6) | 342 | (51.5) |  | 492 | (50.1) | 368 | (47.9) |  |
| Ethnicity (n, %) |  |  |  |  |  |  |  |  |  |  |
| BAME | 629 | (66.3) | 384 | (70.1) | 0.146 | 572 | (59.8) | 426 | (60.4) | 0.847 |
| White | 320 | (33.7) | 164 | (29.9) |  | 384 | (40.2) | 279 | (39.6) |  |
| Employment (n, %) |  |  |  |  |  |  |  |  |  |  |
| Full time | 279 | (32.2) | 161 | (31.9) | 0.955 | 317 | (35.0) | 189 | (28.7) | 0.039 |
| Part time | 224 | (25.9) | 128 | (25.4) |  | 231 | (25.4) | 162 | (24.7) |  |
| Unemployed | 126 | (14.5) | 81 | (16.1) |  | 119 | (13.1) | 96 | (14.7) |  |
| Other | 237 | (27.4) | 134 | (26.6) |  | 241 | (26.5) | 208 | (31.9) |  |
| Occupation (n, %) |  |  |  |  |  |  |  |  |  |  |
| Professional/Managerial | 368 | (56.0) | 204 | (53.7) | 0.003 | 310 | (45.7) | 226 | (48.4) | 0.638 |
| Skilled | 96 | (14.6) | 52 | (13.7) |  | 112 | (16.5) | 80 | (17.1) |  |
| Unskilled | 70 | (10.7) | 22 | (5.8) |  | 79 | (11.7) | 45 | (9.6) |  |
| Other | 123 | (18.7) | 102 | (26.8) |  | 177 | (26.1) | 116 | (24.8) |  |
| Distance to school (n, %) |  |  |  |  |  |  |  |  |  |  |
| Near (≤0.86 km) | 475 | (51.3) | 307 | (55.6) | 0.120 | 351 | (48.3) | 181 | (41.1) | 0.020 |
| Far (>0.86 km) | 451 | (48.7) | 245 | (44.4) |  | 375 | (51.7) | 259 | (58.9) |  |
| Car ownership (n, %) |  |  |  |  |  |  |  |  |  |  |
| Yes | 461 | (54.1) | 42 | (44.7) | 0.103 | 792 | (89.6) | 101 | (94.4) | 0.162 |
| No | 391 | (45.9) | 52 | (55.3) |  | 92 | (10.4) | 6 | (5.6) |  |

**Appendix Table 4 Descriptive characteristics of included and excluded participants in either London or Luton**

* p value refers to independent samples t-tests for continuous variables or Pearson's χ2  tests for categorical variables

**Appendix Table 3 Descriptive characteristics of included and excluded participants in either London or Luton (continued)**

|  | **London** | | | **Luton** | | |
| --- | --- | --- | --- | --- | --- | --- |
|  | **Included (n = 1000)** | **Excluded (n= 664)** | **p-value*** | **Included (n = 982)** | **Excluded (n = 768)** | **p-value*** |

| Crime Quintile (n, %) |  |  |  |  |  |  |  |  |  |  |
| --- | --- | --- | --- | --- | --- | --- | --- | --- | --- | --- |
| 1 | 309 | (31.0) | 174 | (31.4) | 0.010 | 302 | (30.9) | 252 | (35.7) | 0.001 |
| 2 | 301 | (30.2) | 202 | (36.4) |  | 328 | (33.6) | 265 | (37.5) |  |
| 3 | 172 | (17.7) | 95 | (17.1) |  | 237 | (24.3) | 142 | (20.1) |  |
| 4 | 115 | (11.5) | 51 | (9.2) |  | 89 | (9.1) | 42 | (5.9) |  |
| 5 | 101 | (10.1) | 33 | (5.9) |  | 21 | (2.1) | 5 | (0.8) |  |
| IDACI Quintile (n, %) |  |  |  |  |  |  |  |  |  |  |
| 1 | 578 | (57.9) | 291 | (56.0) | 0.009 | 190 | (19.4) | 162 | (23.0) | 0.001 |
| 2 | 269 | (27.0) | 177 | (33.5) |  | 368 | (37.7) | 313 | (44.3) |  |
| 3 | 75 | (7.5) | 32 | (6.1) |  | 282 | (28.9) | 159 | (22.5) |  |
| 4 | 34 | (3.4) | 14 | (2.5) |  | 114 | (11.7) | 58 | (8.2) |  |
| 5 | 42 | (4.2) | 9 | (1.8) |  | 23 | (2.4) | 14 | (2.0) |  |

n: Number; BAME: Black, Asian, and Minority Ethnic; SD: Standard deviation; km: Kilometre

* p value refers to independent samples t-tests for continuous variables or Pearson's χ2  tests for categorical variables

** Car ownership data was only collected at follow-up

Note: Sums of the number of participants with each characteristic may equal the total number of participants if data is missing

**Appendix Table 5 Sensitivity analysis of unadjusted, adjusted, and adjusted multilevel binomial logistic regression models for odds of switching from inactive to active modes and switching from active to inactive modes ‘usually’**

|  | **Switching from inactive to active modes** | | | **Switching from active to inactive modes** | | |
| --- | --- | --- | --- | --- | --- | --- |
| **Predictor variable** | **Unadjusted model** | **Adjusted model** | **Adjusted multilevel model** | **Unadjusted model** | **Adjusted model** | **Adjusted multilevel model** |
|  | OR | OR | OR | OR | OR | OR |
|  | (95% CI) | (95% CI) | (95% CI) | (95% CI) | (95% CI) | (95% CI) |
| Constant | 0.41 | 1.20 | 1.20 | 0.14 | 0.21 | 0.21 |
|  | (0.30 – 0.56) | (0.02 – 108.88) | (0.02 – 85.68) | (0.11 – 0.17) | (0.01 – 4.06) | (0.01 – 4.19) |
| London | 3.00 | 2.81 | 2.81 | 0.17 | 0.12 | 0.13 |
|  | (1.56 – 5.84) | (1.15 – 6.97) | (1.14 – 6.88) | (0.10 – 0.27) | (0.06 – 0.25) | (0.06 – 0.26) |
| Sex (Female) |  | 1.03 | 1.03 |  | 1.03 | 1.03 |
| *Ref: Male* |  | (0.48 – 2.25) | (0.48 – 2.24) |  | (0.63 – 1.69) | (0.62 – 1.69) |
| Age |  | 1.19 | 1.19 |  | 0.90 | 0.90 |
|  |  | (0.73 – 1.95) | (0.73 – 1.94) |  | (0.63 – 1.28) | (0.63 – 1.29) |
| Ethnicity (White) |  | 1.21 | 1.21 |  | 0.58 | 0.58 |
| *Ref: BAME* |  | (0.51 – 2.81) | (0.52 – 2.81) |  | (0.34 – 0.98) | (0.34 – 0.99) |
| Distance to school (Near ≤0.86 km) |  | 2.00 | 2.00 |  | 0.23 | 0.23 |
| *Ref: Far (>0.86 km)* |  | (0.79 – 5.07) | (0.79 – 5.06) |  | (0.13 – 0.39) | (0.13 – 0.40) |
| Vehicle ownership (Yes) |  | 0.06 | 0.06 |  | 5.05 | 5.13 |
| *Ref: No* |  | (0.00 – 0.43) | (0.01 – 0.59) |  | (1.94 – 17.31) | (1.75 – 15.10) |
| Employment (Part-time) |  | 1.31 | 1.31 |  | 1.28 | 1.28 |
| *Ref: Full-time* |  | (0.54 – 3.20) | (0.54 – 3.19) |  | (0.69 – 2.35) | (0.70 – 2.36) |
| Employment (Unemployed) |  | 0.79 | 0.79 |  | 1.16 | 1.17 |
| *Ref: Full-time* |  | (0.27 – 2.18) | (0.28 – 2.22) |  | (0.60 – 2.22) | (0.61 – 2.24) |
| Employment (Other) |  | 0.63 | 0.63 |  | 0.64 | 0.65 |
| *Ref: Full-time* |  | (0.15 – 2.32) | (0.16 – 2.46) |  | (0.24 – 1.57) | (0.25 – 1.67) |
| Crime quintile (linear) |  | 0.96 | 0.96 |  | 1.35 | 1.37 |
|  |  | (0.11 – 5.85) | (0.15 – 6.30) |  | (0.52 – 3.21) | (0.55 – 3.38) |
| IDACI quintile (linear) |  | 0.88 | 0.88 |  | 1.56 | 1.56 |
|  |  | (0.18 – 3.58) | (0.21 – 3.76) |  | (0.66 – 3.56) | (0.67 – 3.64) |
| Observations | 232 | 159 | 159 | 1696 | 1157 | 1157 |
| R^2^ | 0.049 | 0.143 | 0.201 | 0.038 | 0.143 | 0.46 |
| ICC |  |  | 0.00 |  |  | 0.01 |

OR: Odds ratio; 95% CI: 95% Confidence interval; ICC: Intraclass correlation coefficient

**Appendix Table 6 Sensitivity analysis of unadjusted, adjusted, and adjusted multilevel binomial logistic regression models for odds of switching from inactive to active modes and switching from active to inactive modes with re-categorised inactive transport mode variable**

|  | **Switching from inactive to active modes** | | | **Switching from active to inactive modes** | | |
| --- | --- | --- | --- | --- | --- | --- |
| **Predictor variable** | **Unadjusted model** | **Adjusted model** | **Adjusted multilevel model** | **Unadjusted model** | **Adjusted model** | **Adjusted multilevel model** |
|  | OR | OR | OR | OR | OR | OR |
|  | (95% CI) | (95% CI) | (95% CI) | (95% CI) | (95% CI) | (95% CI) |
| Constant | 0.18 | 0.08 | 0.07 | 0.26 | 0.25 | 0.24 |
|  | (0.14-0.24) | (0.00 – 1.98) | (0.00 – 2.01) | (0.21-0.32) | (0.02 – 3.57) | (0.01 – 3.99) |
| London | 3.88 | 4.50 | 4.63 | 0.24 | 0.19 | 0.17 |
|  | (2.46-6.11) | (2.31 – 8.94) | (2.23 – 9.59) | (0.17-0.35) | (0.10 – 0.34) | (0.09 – 0.34) |
| Sex (Female) |  | 1.31 | 1.31 |  | 0.85 | 0.83 |
| *Ref: Male* |  | (0.74 – 2.36) | (0.73 – 2.37) |  | (0.53 – 1.35) | (0.52 – 1.34) |
| Age |  | 1.16 | 1.18 |  | 0.91 | 0.91 |
|  |  | (0.79 – 1.72) | (0.79 – 1.78) |  | (0.67 – 1.24) | (0.66 – 1.26) |
| Ethnicity (White) |  | 2.13 | 2.14 |  | 0.51 | 0.48 |
| *Ref: BAME* |  | (1.15 – 4.01) | (1.12 – 4.06) |  | (0.30 – 0.84) | (0.28 – 0.83) |
| Distance to school (Near ≤0.86 km) |  | 3.97 | 4.07 |  | 0.25 | 0.24 |
| *Ref: Far (>0.86 km)* |  | (2.16 – 7.39) | (2.16 – 7.67) |  | (0.15 – 0.41) | (0.14 – 0.39) |
| Vehicle ownership (Yes) |  | 0.14 | 0.13 |  | 13.49 | 15.03 |
| *Ref: No* |  | (0.05 – 0.34) | (0.05 – 0.35) |  | (5.28 – 45.90) | (5.12 – 44.07) |
| Employment (Part-time) |  | 1.24 | 1.23 |  | 1.37 | 1.31 |
| *Ref: Full-time* |  | (0.60 – 2.52) | (0.59 – 2.54) |  | (0.76 – 2.46) | (0.72 – 2.39) |
| Employment (Unemployed) |  | 1.37 | 1.38 |  | 0.92 | 0.91 |
| *Ref: Full-time* |  | (0.64 – 2.91) | (0.64 – 2.99) |  | (0.48 – 1.72) | (0.47 – 1.74) |
| Employment (Other) |  | 1.44 | 1.51 |  | 0.76 | 0.73 |
| *Ref: Full-time* |  | (0.51 – 3.86) | (0.53 – 4.29) |  | (0.34 – 1.63) | (0.33 – 1.62) |
| IDACI quintile (linear) |  | 0.56 | 0.53 |  | 1.50 | 1.55 |
|  |  | (0.07 – 2.33) | (0.10 – 2.74) |  | (0.72 – 3.02) | (0.72 – 3.33) |
| Crime quintile (linear) |  | 0.52 | 0.56 |  | 2.54 | 2.61 |
|  |  | (0.23 – 1.11) | (0.24 – 1.30) |  | (1.09 – 5.76) | (1.05 – 6.47) |
| Observations | 538 | 373 | 373 | 1390 | 943 | 943 |
| R^2^ | 0.069 | 0.239 | 0.354 | 0.050 | 0.179 | 0.533 |
| ICC |  |  | 0.04 |  |  | 0.07 |

OR: Odds ratio; 95% CI: 95% Confidence interval; ICC: Intraclass correlation coefficient
